# Malignant Ileocolocolic Intussusception in a 19-Year-Old Male

**DOI:** 10.7759/cureus.58937

**Published:** 2024-04-24

**Authors:** Tristan M Palmer, Destino Roman, Westin M Yu, Emilie Fromm, John Stivers

**Affiliations:** 1 Surgery, Lake Erie College of Osteopathic Medicine, Erie, USA; 2 Internal Medicine, Lake Erie College of Osteopathic Medicine, Erie, USA; 3 General Surgery, University of Pittsburgh Medical Center, Erie, USA

**Keywords:** starry-sky appearance, immunohistochemistry staining, r-codox-m/ivac, ileocolocolic intussusception, sporadic burkitt lymphoma

## Abstract

Adult intussusception is an infrequent occurrence typically resulting from an identifiable lead point of a benign or malignant etiology. Here, we present a case of a 19-year-old male who presented to the emergency department with complaints of abdominal pain, intractable nausea, and fluctuations between bloody diarrhea and constipation. These symptoms had begun two months prior and had increased in severity, resulting in significant appetite changes. An abdominal and pelvic computed tomography scan without contrast was obtained, which showed evidence of intussusception of the ileocecum into the transverse colon with resultant small bowel obstruction. The patient underwent an exploratory laparotomy, which resulted in a partial ileocolectomy due to the presence of a 6.8 cm cecal mass with palpable mesenteric lymphadenopathy. The pathologic specimen was identified as Burkitt lymphoma based on a combination of histologic, immunohistochemical, and fluorescence in situ hybridization findings. Currently, the patient is undergoing three cycles of rituximab, cyclophosphamide, vincristine, doxorubicin, high-dose methotrexate, ifosfamide, etoposide, and high-dose cytarabine (R-CODOX-M/IVAC) per Magrath protocol for low-risk Burkitt lymphoma.

## Introduction

Intussusception is defined as the telescoping of a proximal segment of the bowel into the distal adjacent segment, which can lead to intestinal obstruction, inflammation, or ischemia [1]. A diagnosis is more often seen in children, and adult intussusceptions make up 5% of all presentations [2]. Cases of adult intussusceptions are also distinct in etiologies, as 90% of these cases have an identifiable lead point, whereas pediatric cases are often idiopathic [3]. Such lead points include benign and malignant neoplasms, postoperative adhesions, Crohn’s disease, and Meckel’s diverticula, among others [2,4]. While attempted reduction is the treatment of choice in the pediatric population, adult intussusceptions are resolved with surgery (i.e., bowel resection) due to the increased prevalence of an aforementioned lead point in this population. 

Among the colonic neoplasm etiologies, Burkitt lymphoma is a highly malignant and rapidly growing histologic subtype of B-cell non-Hodgkin’s lymphoma. There are three subtypes of Burkitt lymphoma: endemic, sporadic, and immunodeficiency-associated. Sporadic Burkitt lymphoma is the most prevalent of the three in the United States, representing 20-30% of pediatric tumors and approximately 1% of adult non-Hodgkin’s lymphomas [5]. Sporadic Burkitt lymphoma typically presents in extranodal sites, including the gastrointestinal tract, the central nervous system, and bone marrow [6]. When presenting within the gastrointestinal tract, it is typically located within the ileocecal region due to the higher concentration of lymphoid tissue in this area [7]. When present, these extranodal masses have the potential to cause obstruction or, in this case, intussusception. Herein, we report a case of ileocolocolic intussusception in the setting of ileocecal Burkitt lymphoma in a 19-year-old male.

## Case presentation

**Figure 1:**
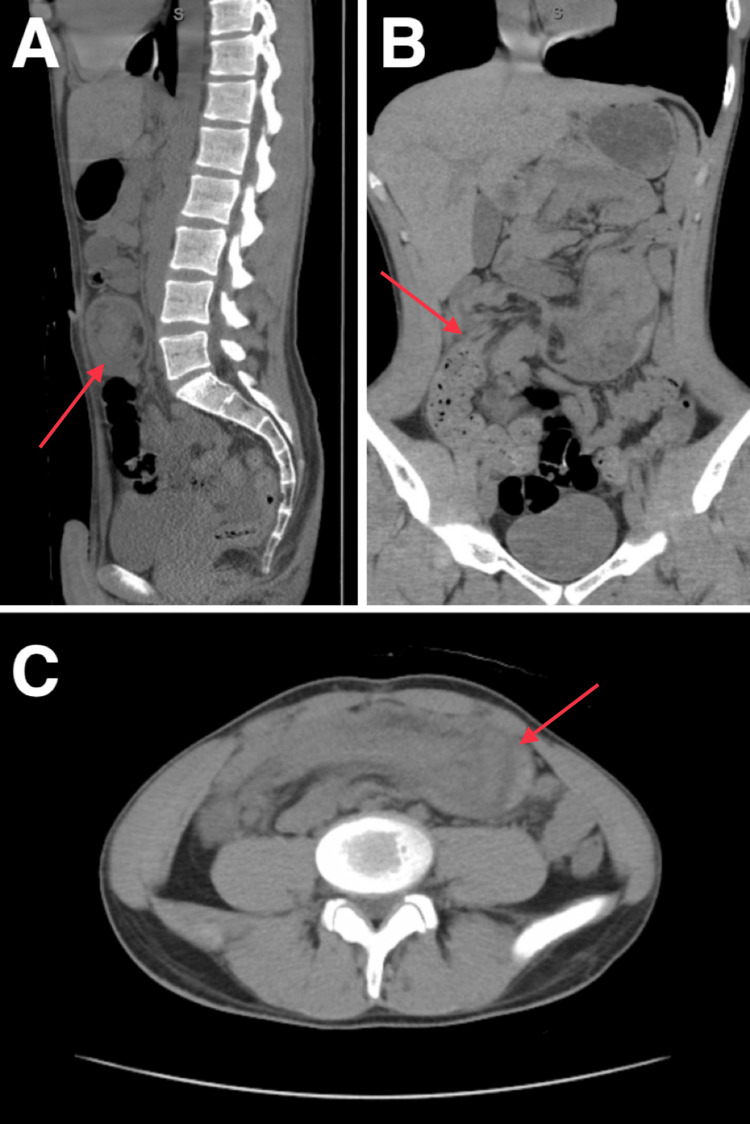
Non-contrast computed tomography of the abdomen and pelvis (A) Coronal view demonstrating intussusception of the ileocecum into the transverse colon with resultant small bowel obstruction. (B) Sagittal view correlating the intussusception finding. (C) Axial view correlating the intussusception finding.

A 19-year-old male without any significant prior medical history presented to the emergency department complaining of intermittent lower abdominal pain accompanied by nausea, fluctuant bloody diarrhea, and constipation for two months duration. His abdominal pain became increasingly severe the day before admission, and he noted an exacerbation with eating. Vital signs were stable, aside from transient tachycardia that reached 122 beats per minute. The patient was given IV fluids, ondansetron, and hydromorphone in the emergency department for symptomatic control. Complete blood count and complete metabolic panels were unremarkable. Computed tomography (CT) of the abdomen and pelvis without contrast revealed intussusception of ileocecum into the transverse colon with resultant small bowel obstruction (Figure 1).

Consequently, acute care surgery was consulted for further management. A physical exam at the time yielded mild left upper quadrant tenderness without distension, rigidity, or guarding, and the patient opted for a trial of nonoperative management with consultation with gastroenterology for potential air enema decompression. However, gastroenterology felt that endoscopic treatment was not advisable at the time, given the patient’s age and duration of symptoms. Furthermore, as the radiology department was not comfortable with performing a gastrografin enema on an adult, the decision was made to proceed with an exploratory laparotomy.

**Figure 2:**
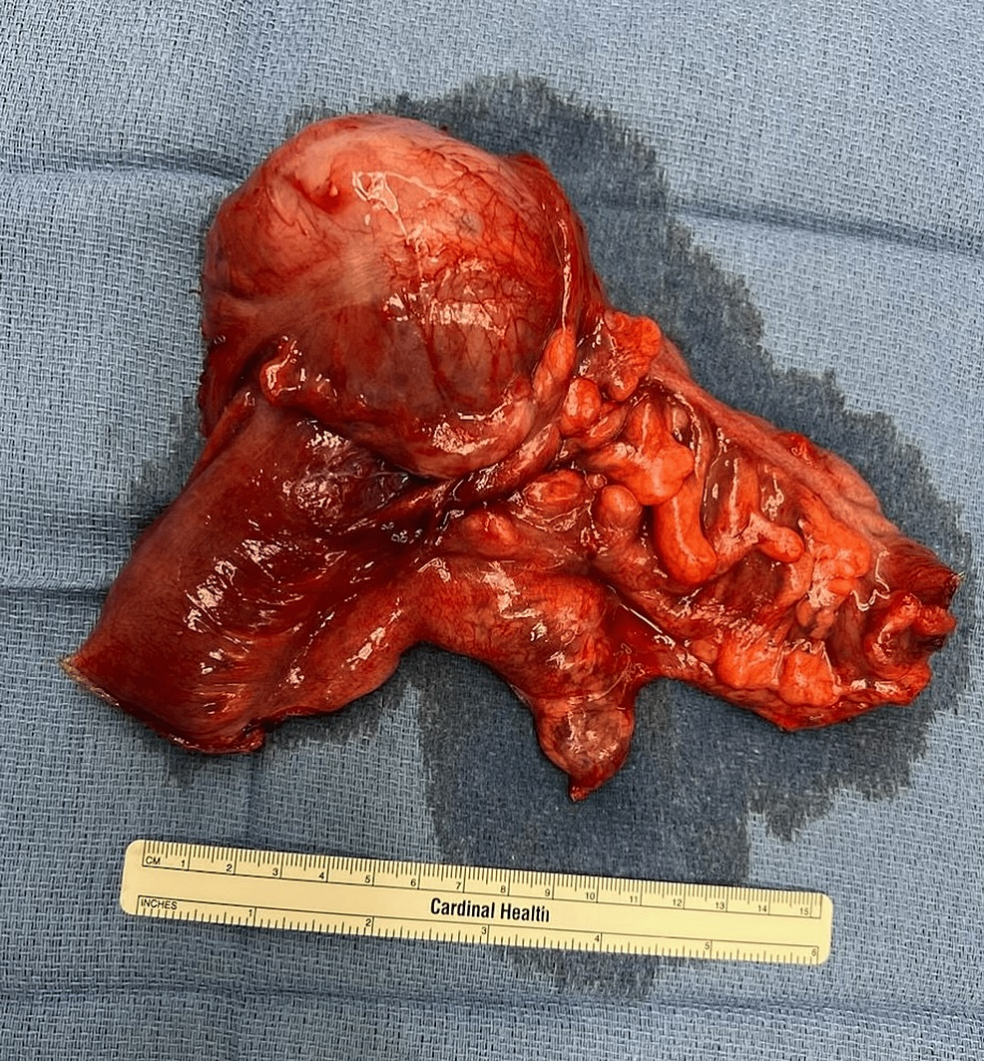
Intra-operative resected specimen with ileocecal mass

Intraoperatively, a palpable mass was identified in the midportion of the transverse colon, consistent with the known intussusception. The right colon was successfully reduced through careful traction and milking distally. However, it was discovered that the patient also had a large cecal mass and several enlarged lymph nodes along the mesenteric basin (Figure 2). Thus, given the concern for malignancy, a formal resection was performed, which comprised approximately 10 cm of colon distal to the cecal mass and 10 cm proximal, including the appendix and terminal ileum. Following the removal of the 28.8 cm long specimen, an ileocolic primary anastomosis was performed, which resulted in no acute complications. 

**Figure 3:**
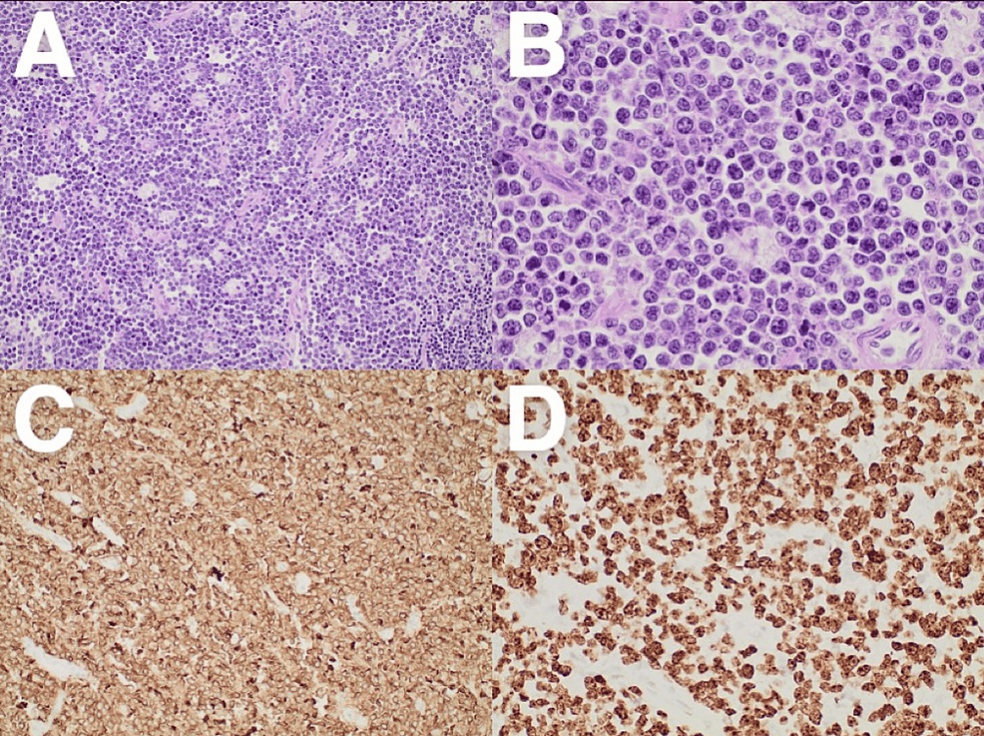
Histologic and immunohistochemistry (IHC) findings of the ileocecal mass (A) 20× magnification demonstrating intermediate-sized B lymphocytes with a “starry sky” appearance. The cells exhibit scant cytoplasm, slightly irregular nuclei, mature chromatin, and multiple small nucleoli. (B) 60× magnification demonstrating high mitotic activity with apoptotic bodies in B lymphocytes. (C) 40× magnification demonstrating CD20 IHC positive B lymphocytes. (D) 40× magnification demonstrating Ki-67 IHC greater than 99% staining in B lymphocytes.

Pathology reported the nodular mass to involve the ileocecal valve and measured it to be 6.8 cm in length by 6.5 cm in circumference by 3 cm in wall thickness. The serosal surface was found to have numerous adhesions, and a denuded focally hemorrhagic mucosal covering was appreciated. Histologic analysis revealed fragments of the intestine with a neoplastic infiltrate, demonstrating a “starry sky pattern” with numerous tingible body macrophages and high mitotic and apoptotic activity (Figure 3). Immunohistochemical analysis revealed the specimen to stain positive for cluster of differentiation 20 (CD20), cluster of differentiation 10 (CD10), and B-cell lymphoma 6 (BCL6) but negative for B-cell lymphoma 2 (BCL2), cyclin D1, cluster of differentiation 3 (CD3), cluster of differentiation 5 (CD5), terminal deoxynucleotidyl transferase (TdT), multiple myeloma oncogene-1 (MUM 1), and LIM domain only 2 (LMO2). Additionally, antigen Kiel 67 (Ki-67) and myelocytomatosis oncogene (MYC) staining were positive in >90% of cells. Chromogenic in situ hybridization with probes for Epstein Barr virus (EBV)-encoded small RNA (EBER) was also positive. Furthermore, high-grade lymphoma fluorescence in situ hybridization panel demonstrated MYC rearrangement but showed no evidence of BCL2 or BCL6 rearrangement. Overall, the culmination of morphologic features and immunohistochemical profile in this case are diagnostic of Burkitt lymphoma. 

The patient was referred to oncology for continued workup, which included obtaining a positron emission tomography (PET)/CT, baseline echo, lumbar puncture, and bone marrow biopsy and aspiration. PET/CT revealed an absence of fluorodeoxyglucose (FDG) avid neoplastic process in the head/neck and chest but demonstrated hypermetabolic soft tissue thickening near the colonic anastomosis, anterior abdominal wall, and omentum, which were indeterminate and believed to likely be the result of postsurgical changes with inflammatory uptake. Additionally, small focal FDG uptake was noted in the left mid-iliac bone but without changes on the CT scan, which was interpreted as benign. Additional studies were determined to be unremarkable. Consequently, the patient was initiated on three cycles of rituximab, cyclophosphamide, vincristine, doxorubicin, high-dose methotrexate, ifosfamide, etoposide, and high-dose cytarabine (R-CODOX-M/IVAC) for low-risk Burkitt lymphoma per Magrath protocol. The patient received filgrastim for growth factor support mid-cycle, acyclovir, fluconazole, and trimethoprim/sulfamethoxazole prophylaxis. Currently, the patient experienced significant but transient toxicity, including severe mucositis, poor oral intake, weight loss, and culture-negative neutropenic fever following the first cycle. The patient has since stabilized and will proceed to the second cycle of treatment.

## Discussion

The nomenclature for intussusception is named by the intussuseptum followed by the intussuscipiens, with the intussuseptum being the proximal part of the bowel that invaginates into the distal portion or intussuscipiens [8]. In our patient, the ileocecal valve was located inside the transverse colon, suggesting an ileocolocolic intussusception. This presentation is an exceedingly rare form of intussusception, with less than 15 reported cases in the literature. Intussusception in adults only accounts for up to 5% of all intussusceptions, as the overwhelming majority are found in children; they also account for only 1% of bowel obstructions [2,8,9]. A specific lead point is identified in 90% of cases, with a neoplasm being the most common in adult intussusceptions, comprising 65% of cases [9]. Chiu et al. suggest that due to the rarity of the ileocolocolic subtype, physicians should have a high suspicion of a neoplastic etiology and a low threshold for resection [9]. The most common type of intussusception found is ileocecal, which is true in both adults and children [2,8]. The combination of the aforementioned factors highlights the exceedingly rare presentation of our patient as well as the need for a broad differential diagnosis when approaching chronic abdominal pain. 

In pediatric patients, the classic triad of symptoms for intussusception consists of paroxysmal abdominal pain, bloody stool, and emesis; in actuality, this triad only presents in 10-20% of patients [10]. Symptoms can vary in intussusception in both adults and children, which commonly include paroxysmal abdominal pain, bloating, nausea, vomiting, bloody diarrhea, fever, alternating bowel habits, and other symptoms of bowel obstruction [2,8]. Symptomatology can also vary depending on the progression of the condition, as bloody diarrhea results from bowel ischemia and sloughing of the necrotic bowel tissue; thus, it is a late symptom that is not seen if diagnosed early. Fever is also regarded as a late symptom, with fever being secondary to bowel perforation and ischemia resulting in sepsis. Our patient presented with characteristic symptoms of intermittent abdominal pain, nausea exacerbated by eating, and alternating bowel patterns, which included bloody diarrhea. This particular combination of symptoms could suggest intussusception, but it is nonspecific, as a variety of other abdominal pathologies may be the culprit.

The gold standard for diagnosing abdominal pathologies following a thorough physical exam is either an ultrasound or CT scan, which serves to narrow down the differential diagnosis. In our patient, a CT scan was performed, which revealed intussusception and subsequent small bowel obstruction. Common CT radiological findings for intussusception include a “stack of coins,” “coil spring,” “sausage,” or “target” lesion appearance, along with other signs such as fat stripes [4,11-13]. A CT scan can also be useful in diagnosing lead points such as Meckel’s diverticula and various neoplasms, along with characteristic small bowel obstruction findings. Ultrasound can be another useful diagnostic imaging technique with a characteristic “sausage” or “target ring” lesion being visible in cases of intussusception [4,11-13]. In our patient, a non-contrast CT was done, which confirmed the diagnosis of intussusception, but a mass was not revealed as the etiology until subsequent exploratory laparotomy.

Neoplasms are the most commonly identified lead point in adult cases of intussusception, with Hong et al. reporting that benign and malignant causes comprise 37.4% and 32.9% of cases, respectively [14]. The most common benign and malignant causes are colonic lipoma and primary adenocarcinoma of the colon, respectively [14,15]. Burkitt lymphoma is an exceptionally rare etiological cause of intussusception, as the most common lymphoma causing intussusception is diffuse large B-cell lymphoma (DLBCL) [16]. However, it is important to note that an estimated 30% of Burkitt lymphoma cases present with intussusception [17]. 

There is no current consensus management protocol for the treatment of Burkitt lymphoma in adults, as many of the current treatment guidelines are extrapolated from pediatric studies. Nonetheless, general treatment guidelines depend on the severity and spread of the disease, and they are comprised of surgical resection, if possible, followed by chemotherapy with rituximab, cyclophosphamide, vincristine, prednisolone, and doxorubicin, among other agents [18,19]. Generally, at least two cycles of chemotherapy with central nervous system prophylaxis are completed, followed by clinical monitoring and re-assessment for residual or recurrent disease [18,19].

## Conclusions

Intussusception in the adult population differs largely from its pediatric counterparts in both the likelihood of a primary cause being present and their standard course of treatment. Herein, we report an exceedingly rare case of intussusception in a 19-year-old male secondary to an extranodal Burkitt lymphoma mass in the ileocecum. The patient underwent resection of the mass with a partial ileocolectomy and is currently undergoing three cycles of rituximab, cyclophosphamide, vincristine, doxorubicin, high-dose methotrexate, ifosfamide, etoposide, and high-dose cytarabine (R-CODOX-M/IVAC) for low-risk Burkitt lymphoma per Magrath protocol. Given the infrequency of both the neoplasm and age of presentation, increased clinical suspicion for malignancy should be given toward intussusception of unknown etiology. This case report and review demonstrate why practitioners must consider malignant etiologies in atypical disease presentations.
